# Fat Face Illusion, or Jastrow Illusion with Faces, in Humans but not in Chimpanzees

**DOI:** 10.1177/2041669515622090

**Published:** 2015-12-14

**Authors:** Masaki Tomonaga

**Affiliations:** Primate Research Institute, Kyoto University, Aichi, Japan

**Keywords:** fat face illusion, Jastrow illusion, face perception, chimpanzees, comparative cognition

## Abstract

When two identical faces are aligned vertically, humans readily perceive the face at the bottom to be fatter than the top one. This phenomenon is called the *fat face illusion*. Furthermore, an apparent similarity has been pointed out between the fat face illusion and the Jastrow illusion. Recent studies have suggested the importance of facial contours and the role of basic-level processing of faces. In the present study, we directly compared the typical Jastrow illusion and fat face illusion in humans and chimpanzees using the same task. Both humans and chimpanzees clearly showed the Jastrow illusion, but only humans perceived the face at the bottom as fatter than the top. Although further examination is necessary, these results might reflect different processing levels of faces between the two species.

Recently, several types of illusions concerning face width or size have been reported. The first was demonstrated by Thompson (2010; Thompson & Wilson, 2012) and called the *fat face thin illusion*: An inverted face is perceived as thinner than an upright face. The second was reported by Araragi, Aotani, and Kitaoka (2012): An upright face is underestimated in size when compared with an inverted face. The third was called the *fat face* illusion: When two identical faces are aligned vertically, the face at the bottom is perceived to be fatter than the one on top (Figure 1(a)). Kitaoka (2006) demonstrated this illusion on his website (Figure 1(a)) and named it the *Jastrow illusion with faces*, partly because of the apparent similarity of stimulus layout and the direction of the illusion (bottom was perceived as longer than the top). Sun et al. (2012, 2013) also studied this phenomenon empirically and found the important role played by face contour in the illusion. Furthermore, Schneider, Hecht, and Carbon (2012) also reported that human observers overestimated body weight (i.e., fatness) for faces photographed from a lower vantage point while underestimated it for faces photographed from a higher vantage point. When comparing these illusions on face width/size, it is interesting to note that the direction of the illusion effect seems to be inconsistent among them. Some researchers reported the importance of internal features (Araragi et al., 2012; Thompson & Wilson, 2012), while others emphasized the role of the outer contour of the face (Sun et al., 2013). One way to address these seemingly contradictory issues is a comparative cognitive approach. There may be implications that emerge from a comparison between humans and another species. To this end, in the present report, we examined the fat face illusion in humans and chimpanzees.

**Figure 1.**
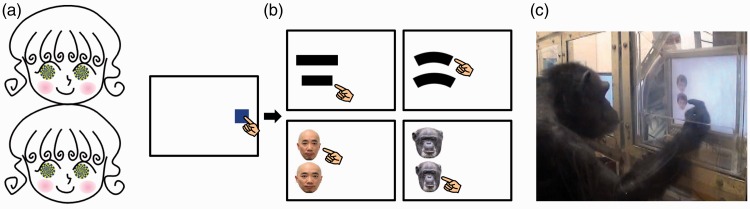
(a) Fat face illusion, or Jastrow illusion with faces (© Akiyoshi Kitaoka, used with permission from the author). The face at the bottom is perceived fatter than the top. (b) Schematic representations of the task. After touching the blue start key, observers were required to touch the *narrower* or *thinner* stimulus of the two. The narrower (thinner) stimulus appeared randomly at the top or bottom position from trial to trial. (c) A chimpanzee participant performing the task.

As Kitaoka (2006) suggested, the vertical alignment of faces is similar to the layout of arc stimuli used in the Jastrow illusion. In this illusion, we often tend to compare the lower arc of the top figure and the upper arc of the bottom figure and, thus, underestimate the size of the top figure. This local comparison account seems also applicable to the fat face illusion; we might compare the contour of the chin of the top face with that of the head of the bottom face.

To directly compare the Jastrow and fat face illusions, we prepared a simple discrimination task as presented in Figure 1(b). Four chimpanzees participated in the experiment (Figure 1(c)). They lived in an enriched environment with nine other chimpanzees in the Primate Research Institute, Kyoto University (KUPRI), and had a long history of participation in perceptual–cognitive tasks (Matsuzawa, 2006; Tomonaga & Imura, 2015). Eight adult humans also participated in the experiments. Four types of stimulus sets were used, as shown in Figure 1(b): rectangles, Jastrow shapes, human faces, and chimpanzee faces. We prepared two different human faces and two different chimpanzee faces. This was to eliminate the possibility that specific cues included in a single face might affect the results. For chimpanzees, these stimuli were transformed from standard stimuli (45 mm in width) by stretching or shortening along the horizontal dimension (width), ranging from 38 to 53 mm for rectangles and Jastrow shapes and from 40 to 50 mm for faces. By pairing these stimuli, we prepared 12 pairs for rectangles and Jastrow shapes and 13 pairs for two types of faces. For humans, stimulus width was ranged from 43 to 47 mm for all stimuli. We prepared 13 pairs for human experiment. Each participant was given a simple discrimination task as shown in Figure 1(b). Each trial began with the presentation of the blue start key at the left center of the screen. After touching this key, two stimuli were presented vertically at the right side of the screen. Participants were required to touch the *narrower* or *thinner* stimulus of the two. The narrower or thinner stimulus appeared randomly at the top or bottom position from trial to trial. Chimpanzees received a food reward (small piece of apple) for every correct choice, while humans were not given any feedback. For chimpanzees, when the width of the stimuli was the same, the response was randomly reinforced with the probability of 50%. Initially each chimpanzee was given baseline training for each condition and then 16 test sessions (48 or 56 trials), while each human participant received 2 sessions (112 trials) for each condition.

The results are shown in Figure 2. We plotted the percentage of responses to the top stimulus as a function of difference ratio of the two stimuli, defined as difference in width between bottom and top stimuli divided by width of the bottom stimuli. The data were fitted to sigmoid functions, and general linear mixed model analyses were conducted using the SPSS software (ver. 19.0 J), in which difference ratio of width was set as fixed effect and subjects and sessions nested in subjects as random effects. Chimpanzees showed very good performances for baseline stimuli (mean accuracy across chimpanzees was 89%). Both chimpanzees and humans showed significant deviation toward choosing the top stimulus in the Jastrow illusion condition (green arrows in Figure 2; chimpanzees, *t*(15) = 9.44, *p* < .001, *r* = .93; humans, *t*(7) = 12.16, *p* < .001, *r* = .98), while they showed neither underestimation nor overestimation with the length of the rectangles (chimpanzees, *t*(15) = .14, ns, *p = *.888, *r* = .04; humans, *t*(7) = 1.99, ns, *p* = .086, *r* = .60). More interestingly, chimpanzees and humans showed clear differences when they judged the width of the faces. Humans showed a very strong *fat face* illusion for both human and chimpanzee faces (human face, *t*(7) = 5.39, *p* = .001, *r* = .90; chimpanzee face, *t*(7) = 6.06, *p* < .001, *r* = .92). The estimated size of the illusion was 3.2%, comparable to a previous study (4%; Sun et al., 2012). However, chimpanzees showed no such bias (human face, *t*(19) = .40, ns, *p* = .697, *r* = .09; chimpanzee face, *t*(19) = 1.51, ns, *p* = .148, *r* = .33).

**Figure 2.**
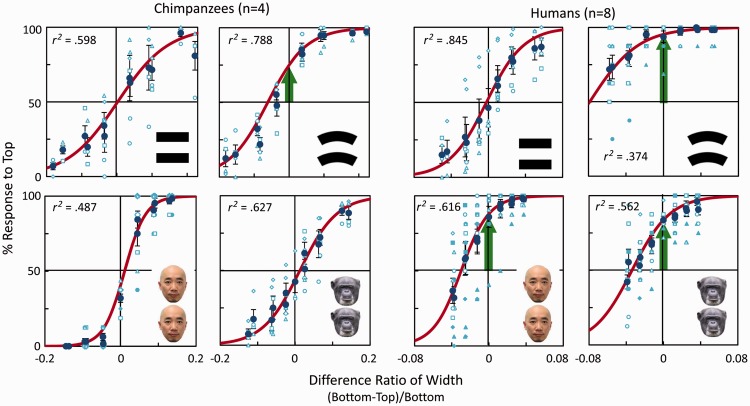
Results of the experiments. The vertical axis shows the percent response to the stimulus at the top. Red curve: sigmoid fitting curve, dark blue circle: mean across participants, light-blue markers: individual data, error bar: standard error. Left panels: chimpanzees, right panels: humans. Green arrows show that the intercept was significantly different from 50%. Error bars show standard errors. Coefficient of determination (*r^2^*) is also shown for each panel.

This is the first reported study to demonstrate the Jastrow illusion in chimpanzees. However, they showed no evidence for the fat face illusion. One possibility is the difference in task procedure between species; chimpanzees were differentially reinforced but not humans. This procedural difference might have affected the present results. However, although this issue should be examined in the future, chimpanzees did show the Jastrow illusion under the identical task for the fat face illusion, suggesting that the current task is sufficient for studying the visual illusion in nonhuman primates. Furthermore, differential-reinforcement tasks have been frequently and successfully used for nonhuman animals (e.g., Fujita, 1997). At best the present results indicate dissociation between the two illusions: The fat face illusion is not explained by a local comparison of the facial contours at least in chimpanzees. Sun et al. (2013) found that the outer contour of the faces had a stronger effect on the fat face illusion than the inner features of the face in humans. They suggested that basic-level processing of the face such as based on outer contour of the face might contribute to the fat face illusion. The outer contour of the face is sufficient for detecting faceness in humans (Hershler & Hochstein, 2005). However, Tomonaga and Imura (2015) showed that an efficient search for the outer contour of the face was not so evident in chimpanzees, consistent with the current results from chimpanzees. Taken together, the results of the present experiment may suggest that the basic-level processing of the face in chimpanzees is different from humans. Further comparative studies on faceness detection and basic-level processing of the face in chimpanzees are necessary to understand the primate origin of the face perception (cf. Tomonaga & Imura, 2009, 2015).
